# A rare case of duodenal diaphragm in an adult during ERCP treatment for choledocholithiasis

**DOI:** 10.1186/s12893-020-00934-1

**Published:** 2020-11-07

**Authors:** Liying Wu, Guofa Jia, Yiheng Hu, Liangsong Zhu, Shuhai Wang

**Affiliations:** Department of Gastroenterology, Huaibei People’s Hospital, No. 66 Huaihai West Road, Huaibei, 235000 Anhui People’s Republic of China

**Keywords:** Acquired, Congenital, Duodenal diaphragm, ERCP, Nsaids

## Abstract

**Background:**

Duodenal Diaphragm in adults is very uncommon, caused by congenital and acquired changes. It is reported that acquired duodenal diaphragm is related to the long-term use of nonsteroidal anti-inflammatory drugs.

**Case summary:**

We report an adult presentation of duodenal diaphragm in a 77-year-old woman, suffered from acute cholangitis and choledocholithiasis. She was performed endoscopic retrograde cholangiopancreatography (ERCP) procedure to remove the stone in common bile duct (CBD). After the stenosis ring dilated by endoscopic balloon dilatation, ERCP procedure was applied, and the CBD stone was removed successfully.

**Conclusion:**

Duodenal diaphragm is difficult to diagnose in clinic. Although the patient in this case had relatively mild symptoms of incomplete upper hemi-abdominal obstruction, these symptoms could be obscured by the emergency acute upper abdominal pain with fever as clinical manifestations of acute cholangitis.

## Background

Duodenal diaphragm usually presents in infancy or childhood with congenital etiology, and it is rare in adults. We report an adult presentation of duodenal diaphragm in a 77-year-old Chinese woman, who underwent endoscopic retrograde cholangiopancreatography (ERCP) procedure to remove the stone in common bile duct (CBD). The occurrence of adult duodenal diaphragm in current case may associate with long-term application of nonsteroidal anti-inflammatory drugs (NSAIDs).

## Case presentation

**Fig. 1:**
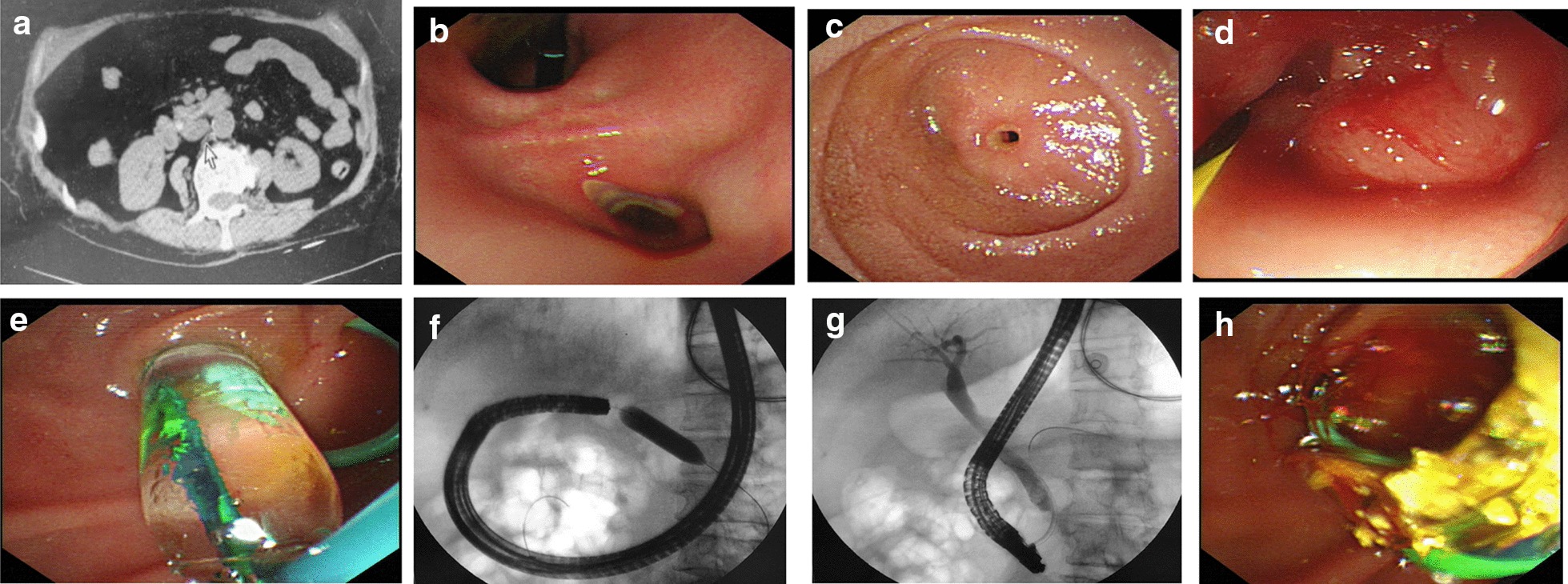
Imaging data for the patient. **a** Abdominal computed tomography showed a high density stone in CBD; **b** endoscopic view of gastric ulcer in pylorus; endoscopic appearance of a duodenal diaphragm with a 2 mm aperture, close view (**c**) and endoscopic dilatation view (**d**); **e** endoscopic view of balloon catheter in situ; **f** radiographic image of balloon dilatation. **g** After dilatation of diaphragm in the lumen, endoscopic tube passed through the stricture, ERCP radiography presented a 1 mm stone in CBD; **h** stone was pulled out through basket

The patient in our case came to Huaibei People’s Hospital emergently accompanied with a 10-h history of acute upper abdominal pain and persistent fever. In order to receive ECRP treatment, she was transferred from local hospital with the diagnosis of acute cholangitis and choledocholithiasis. She had the history of rheumatoid arthritis for 30 years, with irregular treatment by traditional Chinese medicine, NSAIDs and steroids (ibuprofen, indometacin, and prednisone). She also had a history of progressive epigastric fullness for half a year, accompanied with bloating, nausea and occasional emesis after meals, with her body weight decreased for 7.5 kg. Recently, she had three episodes of acute abdominal pain, chills and fever. She have received norfloxacin orally at local clinic, without looking for professional help. Physical examinations revealed a febrile (body temperature 37.6 ℃), and extremely emaciated old woman weighed 34 kg (body mass index 14.5) without jaundice. Abdomen was soft with no tenderness or palpable mass. Her serum albumin level was 22 g/L, and she was anemic with hemoglobin 36 g/L; her white blood cell count was 15.9 × 10^9^/L and serum amylase level was normal. Emergency abdominal CT scan represented that there was a stone in lower part of CBD and a muddy stone in gallbladder (Fig. 1a). According to this critical and emergency situation, she received fluid resuscitation, antibiotic injection and 4-unit red blood cell transfusion.

The next day of admission, the patient signed the consent form of ERCP and gastroscopy procedure, which did not contain the item of biopsy. Then the ERCP procedure was performed on her after her vital signs were stable. Unfortunately, this patient needed for the urgent intervention at that time, thus we did not have enough time to perform the differential diagnostic procedures. Gastroscopy showed an irregular ulceration in the anterior pyloric area (Fig. 1b), and a diaphragm with a 2 mm aperture in the descending part of duodenum (Fig. 1c). The gastroscopic tube cannot pass the aperture. The patient was suffering from critical acute cholangitis, and the failure of ERCP treatment may progress to acute severe cholangitis and sepsis.

We inserted a yellow wire via the stenotic hole to explore whether it was a fistular passway to adjacent organs or the gut (Fig. 1d). By using the X-ray, we found that it connected to the remaining duodenum. The stenotic part of duodenum was short and the diaphragm was soft, so we tried to make the gastroscopic tube pass through the stenotic hole to ensure the successful removal of the CBD stone by balloon catheter dilation. We inserted 10 mm, 12 mm and 14 mm balloon dilatations through stenosis ring respectively (Fig. 1e, f), mild bleeding occurred after the procedure without other complications. Then the gastroscopic tube passed through the stenotic part of duodenum, we switched to the duodenal endoscopy to continue the ERCP procedure. After the balloon dilatation of the papilla, the CBD stone was removed successfully (Fig. 1g, h). After 8 days of hospital stay and medication therapy, her condition was relieved with lab results of hemoglobin 90 g/L and white blood cell count 6.4 × 10^9^/L.

We concluded the final diagnosis as follows: acute cholangitis, choledocholithiasis, gastric ulcer, iron-deficiency anemia, duodenal diaphragm, hypoproteinemia, rheumatoid arthritis and severe malnutrition. Her gastric ulcer, severe anemia and malnutrition might be caused by long term NSAIDs ingestion and food retention due to the upper hemi-abdominal obstruction. Duodenal diaphragm may result from long term NSAIDs usage. We attempted to contact her for a 3-month follow-up after her discharge, but we received no response till now.

## Discussion and conclusion

The presence of duodenal membrane, which is an entity usually identified in infant or children with congenital etiology, is clinically characterized by postprandial vomits of acid and bilious food contents, accompanied by upper hemi-abdominal distention with visible peristalsis and borborygmus. Developmental delay is an important parameter, associated to several conditions such as Down syndrome, prematurity, situs inversus and coexisting extrinsic abnormalities [1–8].

It is reported that acquired duodenal diaphragm is related to long-term use of NSAIDs [9]. Initially it was unknown whether this patient with duodenal diaphragm had congenital etiology or acquired pathogenesis. According to her history consultation, she had a 30-year history of irregular NSAIDs treatment for rheumatoid arthritis. Her symptoms implied the clue for upper hemi-abdominal obstruction, including progressive epigastric fullness, bloating, nausea, and occasional emesis after meals for half a year No similar symptoms were explored before then, therefore, we concluded the diagnosis of acquired duodenal diaphragm.

NSAIDs could contribute to diaphragm-like strictures and ulceration in the small bowel and colon, which typically presents as anemia and bowel obstruction. Diaphragm disease was firstly described by Lang et al. [10]. He reported seven patients with NSAIDs treatment, had developed small bowel strictures resembling perforated diaphragms. The first case of colonic strictures with ulceration was demonstrated by Sheers and Williams [11].

While the symptoms and adverse events are well documented, the detailed pathogenesis of bowel diaphragms remains elusive at present. Going et al. [12] suggested that circumferential ulceration in gut might be the precursor of intestinal diaphragms. The small intestinal diaphragms caused by long-term use of NSAIDs and duodenal diaphragms are similar macroscopically and microscopically [13]. In both cases, diaphragms were formed by the mucosa and submucosa of affected bowels without evidence of scarring or thickening on the serosal surface. Therefore, some researchers [13, 14] speculated that the acquired pathogenesis is related to NSAIDs ingestion. In 1971, Bilbao et al. [15] described a large series of 12 ring-like strictures in the descending duodenum with postbulbar duodenal ulcers. Although some hypotheses of acquired pathogenesis in duodenal diaphragms have been made, it is still unclear whether NSAIDs were an etiologic factor or not.

Several possible etiologies of duodenal strictures should be differentiated, including potassium-induced stricture or neoplastic, ischemic, inflammatory (e.g., Crohn’s disease), and infectious (e.g., tuberculosis) causes, all of which could be excluded by clinical and pathologic grounds.

Many open or laparoscopic procedures have been applied in the surgical treatment of duodenal diaphragm, however, longitudinal duodenotomy with excision of the diaphragm and transverse closure of the duodenotomy is the most prevalent approach for congenital duodenal diaphragm (CDD) treatment [4, 5]. With the advent of endoscopic procedures, the endoscope has become an increasingly powerful therapeutic tool to all types of gastrointestinal strictures. Treatment with different procedures such as endoscopic membranotomy with laser [16], sphincterotome [17], high-frequency-wave snare/cutter [17], hot biopsy forceps [18], insulated-tip diathermic knife [19] and needle knife [20] has been reported. Endoscopic therapy has become popular for a number of reasons: no abdominal incision (scar), no complications such as adhesion development, shorter hospital stays, and sometimes no need of general anesthesia. More invasive endoscopic procedures have been reported, such as cutting the web with an electrocautery device or laser carry risks of perforation, causing excessive bleeding and trauma to the ampulla of Vater. Contrarily, endoscopic dilatation is a relatively safe way with a low risk of mentioned complications, just as we did for the patient in our case.

Altogether, NSAIDs may cause numerous side effects, most seen in the gastrointestinal (GI) tract. NSAID-induced gastrointestinal ulcer is the main complication. Acquired duodenal diaphragm is a very rare malformation, it should be suspected in the cases of abdominal distension and malnutrition. Endoscopy could be used to identify the diagnosis. Endoscopic balloon dilation is a simple and effective method to treat this condition.

## Abbreviations

ERCP: Endoscopic retrograde cholangiopancreatography
CBD: Common bile duct
NSAIDs: Non-steroidal anti-inflammatory drugs
